# Dip TIPS as a Facile and Versatile Method for Fabrication of Polymer Foams with Controlled Shape, Size and Pore Architecture for Bioengineering Applications

**DOI:** 10.1371/journal.pone.0108792

**Published:** 2014-10-02

**Authors:** Naresh Kasoju, Dana Kubies, Marta M. Kumorek, Jan Kříž, Eva Fábryová, Lud'ka Machová, Jana Kovářová, František Rypáček

**Affiliations:** 1 Department of Biomaterials and Bioanalogous Polymer Systems, Institute of Macromolecular Chemistry, Academy of Sciences of the Czech Republic, v.v.i., Prague, Czech Republic; 2 Laboratory of Islets of Langerhans, Institute for Clinical and Experimental Medicine, Prague, Czech Republic; 3 Department of Polymer Processing, Institute of Macromolecular Chemistry, Academy of Sciences of the Czech Republic, v.v.i., Prague, Czech Republic; National University of Ireland, Galway, Ireland

## Abstract

The porous polymer foams act as a template for neotissuegenesis in tissue engineering, and, as a reservoir for cell transplants such as pancreatic islets while simultaneously providing a functional interface with the host body. The fabrication of foams with the controlled shape, size and pore structure is of prime importance in various bioengineering applications. To this end, here we demonstrate a thermally induced phase separation (TIPS) based facile process for the fabrication of polymer foams with a controlled architecture. The setup comprises of a metallic template bar (T), a metallic conducting block (C) and a non-metallic reservoir tube (R), connected in sequence T-C-R. The process hereinafter termed as Dip TIPS, involves the dipping of the T-bar into a polymer solution, followed by filling of the R-tube with a freezing mixture to induce the phase separation of a polymer solution in the immediate vicinity of T-bar; Subsequent free-drying or freeze-extraction steps produced the polymer foams. An easy exchange of the T-bar of a spherical or rectangular shape allowed the fabrication of tubular, open- capsular and flat-sheet shaped foams. A mere change in the quenching time produced the foams with a thickness ranging from hundreds of microns to several millimeters. And, the pore size was conveniently controlled by varying either the polymer concentration or the quenching temperature. Subsequent *in vivo* studies in brown Norway rats for 4-weeks demonstrated the guided cell infiltration and homogenous cell distribution through the polymer matrix, without any fibrous capsule and necrotic core. In conclusion, the results show the “Dip TIPS” as a facile and adaptable process for the fabrication of anisotropic channeled porous polymer foams of various shapes and sizes for potential applications in tissue engineering, cell transplantation and other related fields.

## Introduction

Porous polymer foams are extensively used in various fields of science and technological applications including, but not limited to mechanical, thermal, acoustic and electrical insulations, chemical catalysis, filtration processes and medical devices [1]. In particular, a significant academic and commercial interest has been rising in recent years over the use of polymer foams as scaffolds, along with cells and biological factors, to develop biological substitutes that restore, replace or regenerate defective tissues [2]. For consideration in such bioengineering applications, the scaffolds should (a) be biocompatible, (b) be bioresorbable to provide void volume for neotissuegenesis and remodeling, (c) have an appropriate pore structure for efficient nutrient and metabolite exchange, and (e) provide adequate mechanical or structural stability [2], [3]. Polymers such as degradable polyesters (e.g. polylactide, polyglycolide), silk fibroin, either alone or as composites, and either with or without a functionalization, has been described as biocompatible and bioresorbable materials [4], [5], [6]. However, different tissues/organs in the body have a distinctive architecture in their native states, and thus a scaffold design suitable for all types of tissue engineering is impractical. Therefore, the fabrication of a scaffold with controlled shape, size and pore properties remain a thrust area of research in bioengineering [2].

The physical dimensions such as shape and size of the scaffold play a key role in engineering the desired tissue. For example, the reconstruction of vascular, neural or other tubular tissues requires a hollow tubular scaffold for acting as a physical template and guide neotissuegenesis [7], [8]. In such cases, the tubule thickness and inner lumen diameter should be designed to meet the requirements of the host tissue. The skin or other similar tissue reconstruction strategies demand flat sheet scaffolds [9], [10]. Here also, the thickness should be carefully controlled to avoid the development of any necrotic cores. In addition to regular tubular and flat sheet foams, capsular shaped polymer meshes have been recently reported for use as the matrices for pancreatic islet transplantation applications [11]. Besides, an important criterion that influences the efficiency of tissue reconstruction process is the pore architecture of the scaffold [3]. For instance, the scaffolds with regular isotropic pores often lead to the formation of a necrotic core owing to restriction on the cell penetration and nutrient exchange to the scaffold center caused by a rapid tissue formation on the outer edge of the scaffold [12]. While, the scaffolds with anisotropic pores inherently improve the cell infiltration and nutrient flow, both *in vitro* and *in vivo*, and thus do not lead to any necrotic core formation [12]–[13]. Recent studies also demonstrated that in contrast to the spherical porous scaffolds, the channeled porous scaffolds promote the guided cell infiltration and tissue in-growth and thus yield enriched, homogenously distributed cell population with enhanced functionality [14]–[15].

Various technologies have been widely explored for the fabrication of foams with controlled architectures [3], [16]–[20]. Examples include, (a) solid free-form tools such as three-dimensional (3D) printing, stereo-lithography, laser sintering, (b) porogen involving processes such as gas foaming, phase separation, particulate leaching, and (c) fiber-based techniques such as electrospinning or fiber bonding [3], [16]–[20]. Amongst all, the phase separation process, particularly the thermally induced phase separation (TIPS), was efficient in the preparation of interconnected porous foams [21]. Additionally, by applying a unidirectional thermal gradient, it was possible to obtain the anisotropic channeled porous scaffold [21], [22]. The standard TIPS setups were used to prepare regular 3D foams with channeled pores, but without the limitation on the final shape and size [22]. The scaffolds in the form of hollow fibers or tubes (e.g. for reconstruction of vascular vessels or other tubular tissues) have also been fabricated [23], [24]. However, the developed methods were based on complex setups involving (i) several thermal conducting and insulating components, (ii) multiple systems to obtain differently shaped foams, and (iii) multiple adjustments to vary the foam thickness. The pores were usually axially oriented to the tube lumen and the outer surface of the foams often exhibited less-/non- porous skin that restricted the cell infiltration [22]–[26].

Here we demonstrate a TIPS-based efficient, facile and adaptable methodology, hereby termed as Dip TIPS, to obtain the polymer foams with a controlled shape, size and pore design. We tested the versatility of the method to yield the foams with (a) variable shapes such as tubes, open-end capsules and flat 3D sheets, (b) variable inner lumen diameters in the case of tubes and capsules, (c) variable thickness, ranging from hundreds of microns to several millimeters and with (d) controlled anisotropic interconnected channeled pores. The systematic investigations were performed to determine first the influence of polymer properties (such as polymer type, molecular weight, concentration), then process parameters (such as quenching temperature and time, coarsening duration) and finally mold properties (such as template diameter) on the final foam architecture. The feasibility of the scaffolds for use in bioengineering applications, such as guided tissue engineering, was tested by the *in vivo* implantation in the male brown Norway rats followed by the histochemical and immuno-histochemical analysis of the excised implants.

## Materials and Methods

### Materials

We purchased L-lactide, ε-caprolactone, Tin(II) 2-ethylhexanoate, phosphate buffer saline (PBS) tablets from Sigma-Aldrich, Czech Republic, paraffin (histowax 56–58°C) from Bamed s.r.o., Czech Republic, hematoxylin and eosin from Roche s.r.o., Czech Republic, anti-CD31(cluster of differentiation 31, or also known as platelet endothelial cell adhesion molecule-1) antibody from Acris Antibody GmbH, Germany. All other reagents and chemicals were obtained from P-Lab a.s., Lach-Ner s.r.o. and Sigma-Aldrich Czech Republic, and were used as received.

### Synthesis of polymers

High molecular weight poly(L-lactide) (PLA), poly(ε-caprolactone) (PCL) and poly(L-lactide-*co*-ε-caprolactone) (PLCL) were synthesized by ring-opening polymerization or co-polymerization of the corresponding monomers (L-lactide, ε-caprolactone) in the presence of Tin(II) 2-ethylhexanoate as a catalyst in bulk as described previously [27], [28]. The molecular weight of the polymers was determined using a gel permeation chromatography (Waters Corporation) and the details are as follows: PLA: *M*_w_ = 220 000 g/mol and *M*_n_ = 98 000 g/mol; PCL: *M*_w_ = 120 000 g/mol and *M*_n_ = 80 000 g/mol; PLCL300: *M*_w_ = 316 000 \g/mol and *M*_n_ = 120 000 g/mol; PLCL150: *M*_w_ = 162 000 g/mol and *M*_n_ = 57 000 g/mol. The content of ε-caprolactone in both PLCL copolymers was found to be 7% mol as analyzed by the ^1^H nuclear magnetic resonance analysis (DPX 300, Bruker).

### Dip TIPS setup

**Figure 1:**
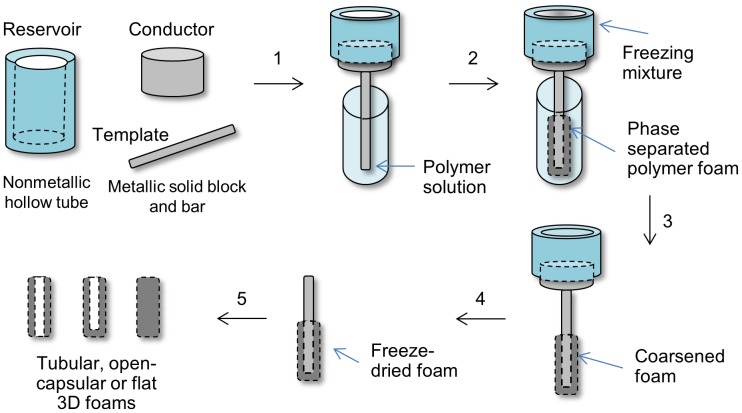
Dip TIPS scheme. An illustration of the essential components of the setup (*reservoir*, *conductor* and *template*) and the sequential steps involved in the Dip TIPS process (1: assembling, 2: quenching, 3: coarsening, 4: freeze-drying and 5: collection of the foam).

Figure 1 shows the experimental setup for the fabrication of polymer foams by dipping the template bar of a particular size and shape into a polymer solution followed by controlled cooling. The setup consists of (a) a thermally conductive metal template bar with variable macro-shapes and dimensions (hereby referred to as “*template*”, dimensions: a cylindrical bar for tubular or capsular foams: 2, 3 or 4 × 40 mm diameter and height respectively; a planar bar for flat 3D foams: 3 × 10 × 40 mm thickness, width and height respectively), (b) a thermally conductive metallic solid cylindrical block with pre-defined dimensions (hereby referred to as “*conductor*”, 30 × 30 mm diameter and height respectively), along with (c) a thermally low/non-conductive non-metallic hollow tube used as a reservoir for the cooling mixture (hereby referred to as “*reservoir*”, 30 × 120 mm diameter and height respectively). The *template* was attached at one end to the *conductor* block that was in turn bolted into the *reservoir* tube (*template* → *conductor* → *reservoir*). The whole setup was arranged properly onto a laboratory stand with the help of appropriate holders.

### Dip TIPS process

The fabrication process is schematically presented in Figure 1. The *template* and the *conductor* maintained in a water bath at 40°C were taken out and wiped with tissue paper and were then immediately assembled together along with the *reservoir*. First, the *template* was dipped into the polymer solution at ambient temperature. Then, the *reservoir* tube was filled with an appropriate cooling mixture to quench the polymer solution in the immediate vicinity of the *template*. After a preset quenching time, the *template* with the frozen polymer foam was removed from the polymer solution and was allowed to coarsen for a defined period. The coarsened foams were then freeze-dried under vacuum in the dry ice/ethanol bath for initial 1-2 days and then in ambient temperature for at least another 1 day. Alternatively, the coarsened foams were subjected to the freeze-extraction process by immersing into pre-cooled ethanol for 2–3 days with intermittent solvent exchange. The scaffolds were then collected from the *template* and kept in a desiccator until further use.

Ethanol with a controlled addition of dry ice, dry ice/ethanol and liquid nitrogen baths were used as the cooling mixtures to achieve the temperatures of −25, −80 and −196°C, respectively. The experimental conditions were tested in the order as they are listed in Table 1. After each experiment, the tested parameter leading to appropriate scaffold parameters was fixed for further test. First, the effect of the polymer type was tested and the polymer giving the optimal results was selected for the following experiment. Then, the effect of molecular weight was tested and the polymer with appropriate results was selected for the test of the polymer concentration. Having selected polymer parameters, we likewise performed the systematic experiments to optimize various Dip TIPS process conditions.

**Table 1:** Parameters under Dip TIPS study with the information about tested variables and other constant conditions followed* (n = 6).

Variable parameter	Details	Constant experimental conditions
Polymer type	PLA, PCL, **PLCL**	Polymer concentration: 5% (w/v)
		Quenching temperature: −80°C
		Quenching time: 30 s
		Coarsening time: None
Polymer molecular weight	162000 g/mol (PLCL150)	Polymer concentration: 5% (w/v)
	316000 g/mol (**PLCL300**)	Quenching temperature: −80°C
		Quenching time: 30 s
		Coarsening time: None
Polymer concentration	3, **5**, 7, 10% (w/v)	Polymer type: PLCL300
		Quenching temperature: −80°C
		Quenching time: 30 s
		Coarsening time: None
Quenching temperature	−25, **−80**, −196°C	Polymer type: PLCL300
		Polymer concentration: 5% (w/v)
		Quenching time: 30 s
		Coarsening time: None
Quenching time	15, **30**, 45, 60 s	Polymer type: PLCL300
		Polymer concentration: 5% (w/v)
		Quenching temp: −80°C
		Coarsening time: None
Coarsening duration	**0**, 30, 60, 120 min	Polymer type: PLCL300
		Polymer concentration: 3% (w/v)
		Quenching temperature: −25°C
		Quenching time: 30 s
Mold diameter (for tubular and open-end capsular foams)	2, **3**, 4 mm	Polymer type: PLCL300
		Polymer concentration: 5% (w/v)
		Quenching temperature: −80°C
		Quenching time: 30 s
		Coarsening time: None

* First, the effect of the polymer type was tested and the polymer giving the optimal results was selected for the subsequent experiment. Next, the effect of molecular weight was tested and the polymer with best results was selected for the test of the polymer concentration. Having selected polymer parameters, we then progressively optimized the process conditions. The parameter fixed after each step is marked in bold font.

### Scaffold characterization

#### Morphological properties

The morphological features of the pores and the pore patterns were examined by a scanning electron microscope (SEM, Vega, Tescan; n = 6). Typically, a foam was cut carefully with a fine razor to expose the outer and inner surfaces, and the cross and longitudinal sections. Prior to the analysis, all samples were coated with Platinum in a sputter coater (SCD050, Leica Microsystems) for 120 s at 40 mA current and pressure below 10^−1^ mBar. The diameter of the pores was determined from SEM images using *Image J* freeware. At least 25 random pores were measured from each image to calculate the average diameter.

#### Porosity and surface area analysis

The PLCL300 foams prepared from a 5% w/v solution by quenching at −25, −80 and −196°C for 30 seconds were analyzed to explore the typical pore properties and their relationship with the processing conditions (n = 3). First, to determine the open-porous character, the foams were subjected to mercury intrusion porosimetry analysis (Pascal 140 and 440, Thermo Finigan, Rodano, Italy). A mercury tension of 480 mN/m and a contact angle of 141.3° were imposed for all measurements. The applied pressure of mercury was in the range of 0.01–400 MPa allowing the determination of meso- and macro- pores from 4 nm to 116 µm. The pore volume and the most frequent pore diameter were calculated from the cumulative pore volume curves by the Pascal program with the use of Washburn equation under the assumption of the cylindrical pore model [29]. The porosity was calculated according to the equation (1), where *V* is the cumulative pore volume (ml/g) and ρ is density of the used polymer. 
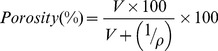
(1)


The specific surface area of the foams was measured by a gas adsorption technique using nitrogen as the adsorbate and liquid nitrogen (77 K) as a cooling medium (Gemini VII 2390, Micromeritics Instruments Corp.). The surface area was calculated from the Brunauer-Emmett-Teller (BET) plot of the adsorption/desorption isotherm using the software supplied with the instrument. Prior to the porosimetry and surface area analysis, the samples were dried under vacuum at 40°C overnight. For all calculations, the polymer density (ρ) of 1.3 g/cm^3^ was used.

#### Thermal properties characterization

The PLC300 foams prepared from a 5% w/v solution by quenching at −25, −80 and −196°C for 30 seconds were subjected to differential scanning calorimetry (DSC, Pyris 1, Perkin-Elmer) analysis (n = 3). The degree of crystallinity was calculated following the equation (2), where, *X_c_* (%) refers to the percentage of crystallinity, Δ*H*_f_ refers to the heat of fusion of the sample, while, Δ*H*_f_° refers to the theoretical heat of fusion of 100% crystalline sample [30]. Due to the lack of Δ*H*_f_° value of the copolymer under study, the literature proposed to consider as Δ*H*_f_° the Δ*H*_f_° value of the major portion of the copolymer [30], [31]. Accordingly, in the current study, since the PLCL300 has 93% (w/w) of lactide content, the heat of fusion of pure PLA (i.e., 93 J/g) was considered as the Δ*H*_f_° value.



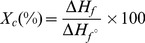
(2)


### *In vivo* studies

#### Ethics statement

This study was carried out in strict accordance with the recommendations for the care and use of laboratory animals of the Institute for Clinical and Experimental Medicine (ICEM), Prague, Czech Republic. The Animal Care Committee of the ICEM and the Ministry of Health, Czech Republic approved all the protocols related to this study. Animals were kept according to the European Convention on Animal Care in a controlled temperature, humidity and 12/12 light/dark regimen, with a free access to the sterile food pellets and water. All surgical procedures were done under the total anesthesia induced by a mixture of medetomidine and ketamine injected intramuscularly. At the end of the study, the test samples were excised from animals under the general anesthesia induced by a mixture of medetomidine and ketamine injected intramuscularly and subsequently, the animals were euthanized using exsanguination. During the entire study, all efforts were made to minimize suffering for the animals.

#### *In vivo* implantation

The PLCL300 foams prepared with a convenient pore size and physical strength prepared at the optimized conditions (5% w/v in dioxane, quenched at −80°C for 30 s) were used in the *in vivo* study. The control samples were products of ELLA-CS, Ltd. Czech Republic and were made from monofilament polydioxanone fibers by knitting, with a fiber thickness of ∼200 µm and a mesh size between 500 to 700 µm. Prior to the implantation, all scaffolds were sterilized by 70% ethanol treatment for 1 h and were then thoroughly washed with sterile distilled water. The foams were implanted into the greater omentum of model brown Norway rats (male, aged between 2–3 months, 250–270 g, n = 6). After placement of implants, the incisions were sutured in two levels with 4-0 vicryl absorbable sutures.

#### Histochemical and immuno-histochemical staining

After four weeks of implantation, the rats were euthanized and the implants were harvested. For histological analysis, the implants were washed in PBS, fixed with 4% formaldehyde in PBS overnight, dehydrated through a graded series of ethanol, embedded in paraffin and sectioned at a thickness of 5 µm. The sections were then de-paraffinized, rehydrated with a graded series of ethanol and stained with the following stains as per the standard protocols: hematoxylin and eosin (H&E, to display the cytoplasmic and nuclear features of the tissue), masson's trichrome (TRI, to display extracellular matrix components, particularly collagen) and anti-CD31 antibody (CD31, to display the presence of vascular endothelial cells).

### Sample size and statistical analysis

The sample size in SEM analysis and *in vivo* studies was 6, and in porosimetry, BET and DSC analysis was 3. The data presented was a representative of respective ‘n’. The quantitative values were averaged and expressed as mean ± standard deviation (SD). The statistical differences were determined by the Student's t-test and the differences were considered as statistically significant at P<0.01. The quantitative data was also subjected to Pearson's correlation analysis to deduce the relationship between two properties. The coefficients (R) of −1 and +1 were considered to represent the inverse and direct correlation respectively, while the 0 represents no correlation. The closer the coefficient is to either −1 or +1, the stronger the correlation between the variables.

## Results and Discussion

### Typical features of pore architecture

**Figure 2:**
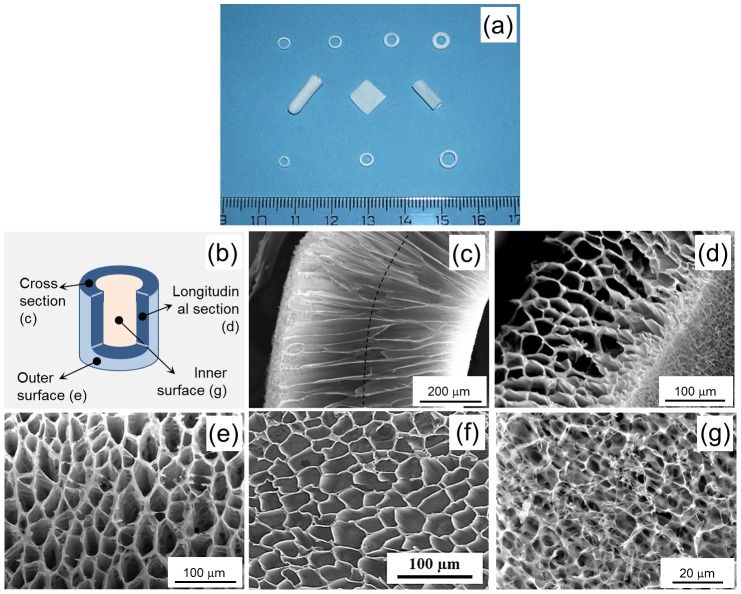
Macro- and micro- morphological features of the foams. (a) gross appearance of the open-end capsular, tubular, flat 3D porous PLCL300 scaffolds (5% w/v, quenched at −80°C) with variable foam thickness and inner lumen diameters; (b) schematic of different sections of the scaffold analyzed under SEM; typical SEM images of (c) cross section of the scaffold showing the lengthwise cut elongated pores in direction of the applied thermal gradient (pore walls and overall channeled character of pores are clearly visible), (d) longitudinal section of the scaffold showing interconnected open-cells between particular channels, and (e) outer surface and (g) inner surface of the scaffold revealing the anisotropic nature of the pores. Panel (f) is the middle section of the foam's cross section (black dotted line in the panel c) showing an internal honey-comb like pore architecture.

The gross appearance and SEM images of the foams are presented in Figure 2. As may be seen, the current methodology readily enabled the fabrication of foams in shapes such as open-end capsules, tubules and flat 3D sheets, and with variable foam thickness and inner lumen diameters (Figure 2a). The cross section image (Figure 2c) shows the lengthwise cut pores made by the solvent crystals formed perpendicularly to the lumen. The pores are long and nearly continuous from one end to the other with intermittent branches also indicating the channeled structure. The anisotropic nature of the pores with a gradual increase in the pore size from inside to outside is evident as well. A high thickness of the analyzed sample did not show the open-cells in this section. However, the longitudinal section of the tubular scaffold clearly depicted its open-porous and interconnected nature (Figure 2d). The images of the scaffold surface showed bigger pores on the outer surface of the foam (Figure 2e) and relatively smaller pores on the inner surface that was in direct-contact with the *template* (Figure 2g), and thus confirmed an anisotropic pore structure. Further, the inner pores were found to be randomly organized, whereas, the outer pores exhibited a well-organized honeycomb shaped channeled network. A vertical cut in the middle of the foam revealed the well-organized honeycomb shaped pore structure under the outer surface also (Figure 2f). Remarkably, the overall observations revealed that the channels were without any transverse cross walls, as in contrast to previous reports describing the foams with a significant amount of transverse ladder-like cross walls that would plausibly obstruct the guided cell infiltration and nutrient flow [24], [32].

### Influence of polymer properties on pore architecture

#### Polymer type

**Figure 3:**
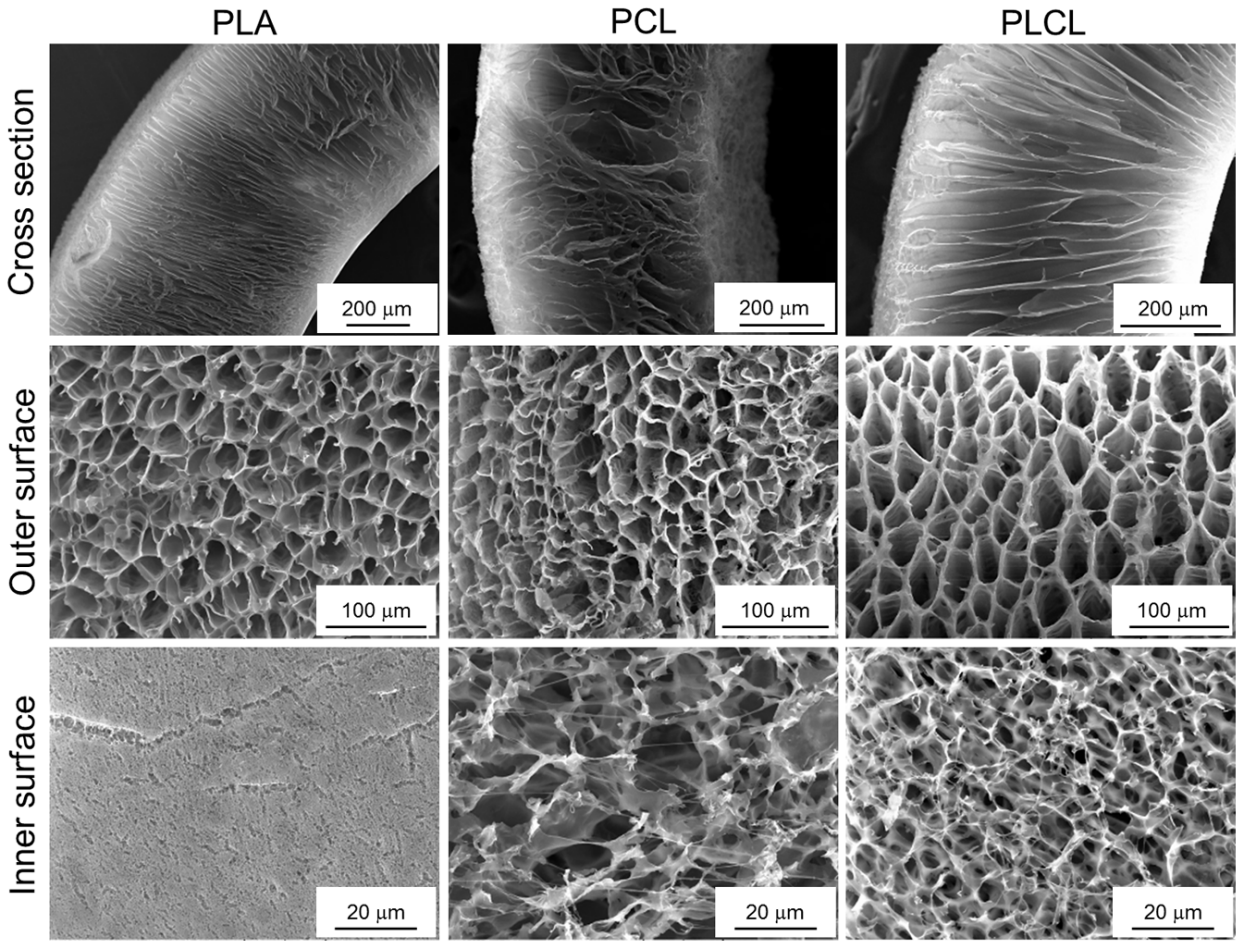
Influence of the polymer type. SEM images of the cross section, the outer and inner surfaces of the foams prepared from PLA, PCL and PLCL300 (5% w/v, quenched at −80°C for 30 s) revealed the relatively superior pore morphology without any micro-cracks of the PLCL foam as compared to that of pure PLA and PCL foams.

To understand the influence of the polymer type on pore architecture, we prepared PLA, PCL and PLCL foams under same experimental conditions (Table 1). The SEM images showing the pore morphology of foams are presented in Figure 3. It was observed that the PCL foam resulted in a poorly-ordered pore structure and was too soft and elastic to handle. This could be attributed majorly to low glass transition temperature (*T_g_*, −60°C) of the polymer and its consequent impact on the overall phase separation phenomenon at the applied quenching temperature of −80°C [33]. On the other hand, PLA with *T_g_* of 60°C successfully yielded the well-oriented foam at the same quenching temperature. However, a detailed examination of the inner surface of the foam that was in direct-contact with the *template* revealed micro-cracks and a significantly reduced porosity. The development of such micro-cracks (or often referred to as crazes) could be attributed to the inherent chain stiffness of PLA [25], [34]. In contrast, the PLCL foam with a PLA:PCL ratio of 93∶7 mol/mol exhibited a well-ordered pore morphology without any crazes and with tough yet flexible mechanical properties compared to relatively brittle PLA scaffolds. Such improved features could be attributed to the polymer properties (molecular weight, chain stiffness and *T_g_* value of 30°C), and the final quench depth (the temperature difference between the melting point of the solvent or *T_g_* of the polymer or the cloud point of the polymer solution and the applied quenching temperature). Thus, it was evident that in principle the current methodology could be applicable to any polymer type, but, inherent properties of the polymer would influence the pore architecture and mechanical strength of the resulting foam. Since PLCL was found to yield favorable features of the foams, further studies were carried out using this polymer.

#### Polymer molecular weight

**Figure 4:**
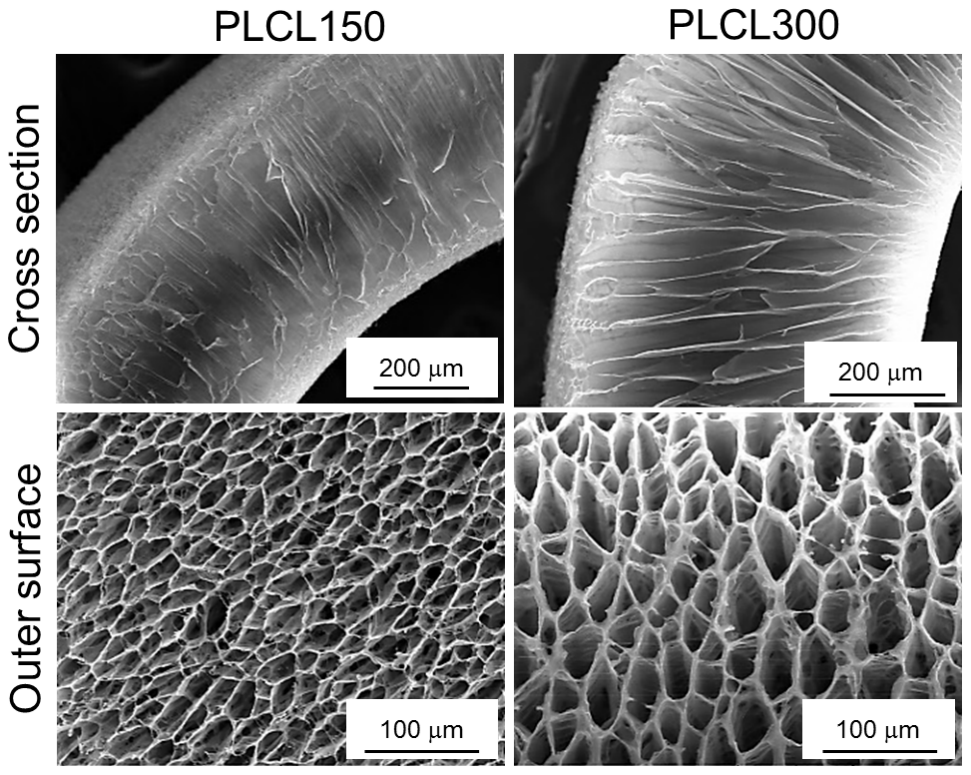
Influence of the polymer molecular weight. SEM images of the cross section and the outer surface of the foams prepared from PLCL solution (5% w/v, quenched at −80°C for 30 s) of variable molecular weights showed the well-organized pore morphology and increased overall foam thickness in PLCL300 (*M*_w_ = 316,000 g/mol and *M*_n_ = 120,000 g/mol) as compared to that of PLCL150 (*M*_w_ = 162,000 g/mol and *M*_n_ = 57,000 g/mol).

To investigate the effects of the polymer molecular weight on the pore morphology, we prepared the foams from PLCL of two different molecular weights, i.e. *M*_w_ = 316 000 g/mol (PLCL300) and *M*_w_ = 162 000 g/mol (PLCL150) under same experimental conditions (Table 1). SEM images of the cross section and outer surface of the scaffolds are presented in Figure 4. In contrast to the PLCL150 foams, the PLCL300 foams had well-organized pore structure with significantly thicker pore walls and enhanced toughness. Also, the PLCL300 foams exhibited higher overall foam thickness (612±22 µm) as compared to that of PLCL150 (469±25 µm). Thus, despite the same concentration and phase separation conditions, the order of the pores, the pore wall thickness, the overall foam thickness and toughness (by physical examination) were directly proportional to the increased polymer molecular weight, while, the pore size was inversely related. The observations comply with those of previous studies of PLA-based TIPS foams, where the increasing polymer molecular weight was suggested to reduce the polymer-solvent interactions and thereby to increase the cloud point or the freezing temperature [35], [36]. Additionally, the increasing viscosity along with increasing polymer-rich phase were to be accounted, at least partially, for the enhanced order in the pore structure of foams prepared from higher molecular weights [35], [36]. Due to the favorable features of the foams, PLCL300 was used for following investigations.

#### Polymer concentration

**Figure 5:**
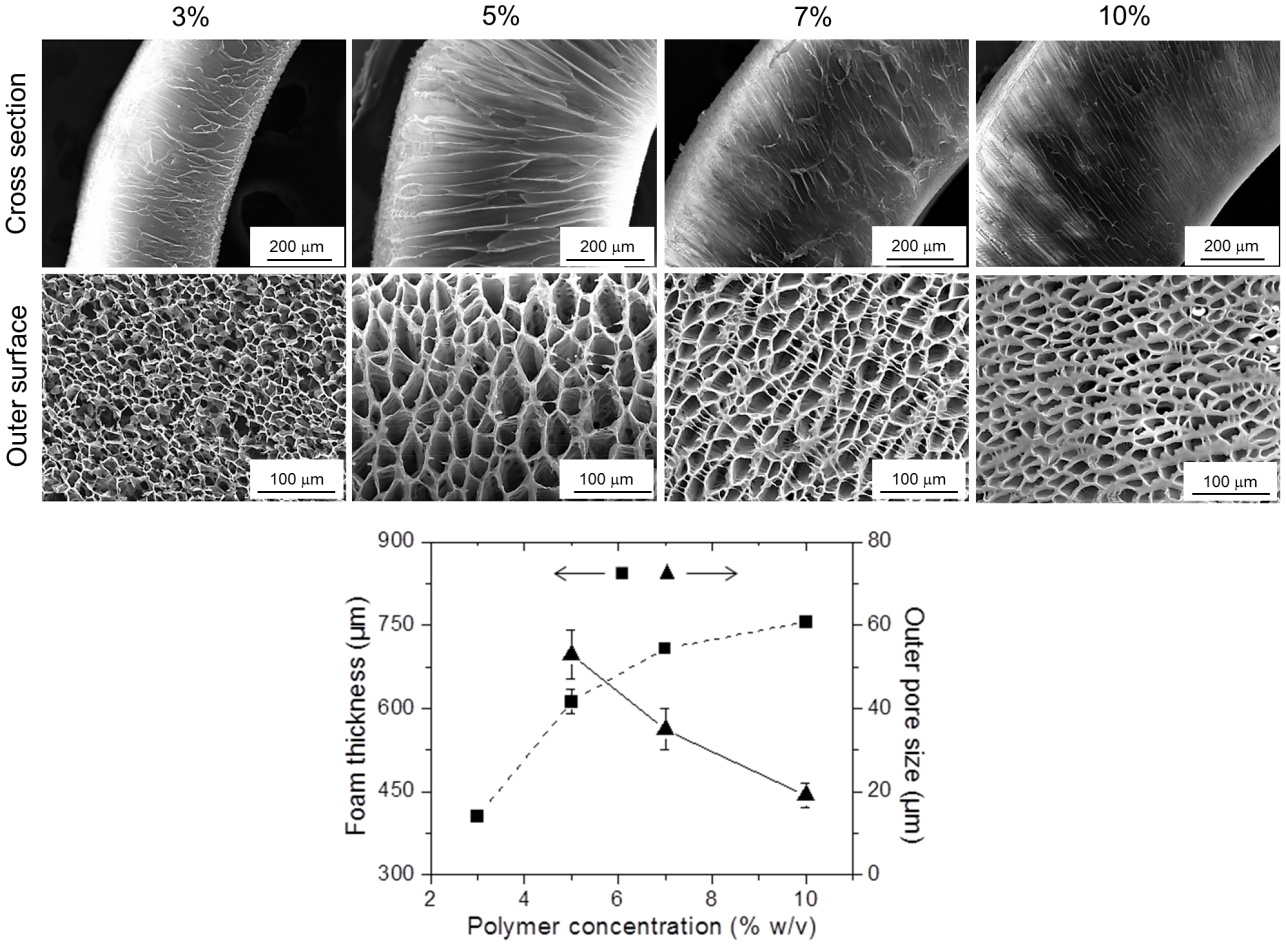
Influence of the polymer concentration. SEM images of the cross section and the outer surface of the PLCL300 scaffolds prepared from variable concentration (3, 5, 7 and 10% w/v, quenched at −80°C for 30 s) demonstrated that the pore size was inversely proportional, and the pore wall thickness and the overall foam thickness was directly proportional to increase in the polymer concentration. Measurements were performed by *Image J* (*n* = 25). The differences in the foam thickness or the pore size between any two groups were found to be statistically significant. The correlation coefficients between the polymer concentration and the outer pore size and the foam thickness were calculated to be −0.99 and +0.92, respectively.

The effect of the polymer concentration on the foam morphology was investigated using the PLCL300 polymer. Four concentrations were tested: 3, 5, 7 and 10% (w/v), respectively. Figure 5 presents SEM images of cross sections and the outer surface of PLC300 foams along with a plot displaying the trend in the foam thickness and the outer pore size. As evident, the foams prepared from 3% (w/v) PLCL solution exhibited irregular and undefined pore architecture mainly due to deficiency of a polymer-rich phase. In contrast, the foams with higher polymer content demonstrated the well-organized channeled pores. There was a gradual decrease in the outer pore size from 53±6 to 35±5 to 19±3 µm with an increasing polymer concentration from 5, 7 to 10%, respectively. SEM observations of the outer pore morphology also revealed a gradual thickening of the pore wall in direct relation to the polymer concentration. And, the pores on the inner side which was in a direct contact with the *template* were randomly organized with the size ranging between 5-10 µm (Figure 2g), and were following a decreasing trend with respect to the increasing polymer concentration (data not presented). As reported previously, such decrease in the pore size was attributed to reduction in the solvent crystallite phase that was more often interrupted by the polymer phase. While, an increase in the pore wall thickness was due to the possible spinodal phase separation including the exclusion of the solvent from the polymer phase to stabilize the overall system in terms of thermodynamic interfacial energy [35], [32], [37], [38]. In the current study, SEM images of cross sections also showed a clear increase in the overall foam thickness of 405±9, 612±22, 708±8 and finally 756±9 µm with increasing polymer concentration from 3, 5, 7 to 10% (w/v) respectively. This could be attributed to the gradual decrease in the solvent phase with an increase in the polymer concentration and its subsequent influence on the overall phase separation process under the same applied quenching temperature. Besides, as obvious, the physical examination of the foams suggested an increased toughness with an increase in the polymer content. To optimize Dip TIPS process conditions, the 5% (w/v) solution of PLCL300 was selected for the following experiments.

### Influence of processing conditions on pore architecture

#### Quenching temperature

**Figure 6:**
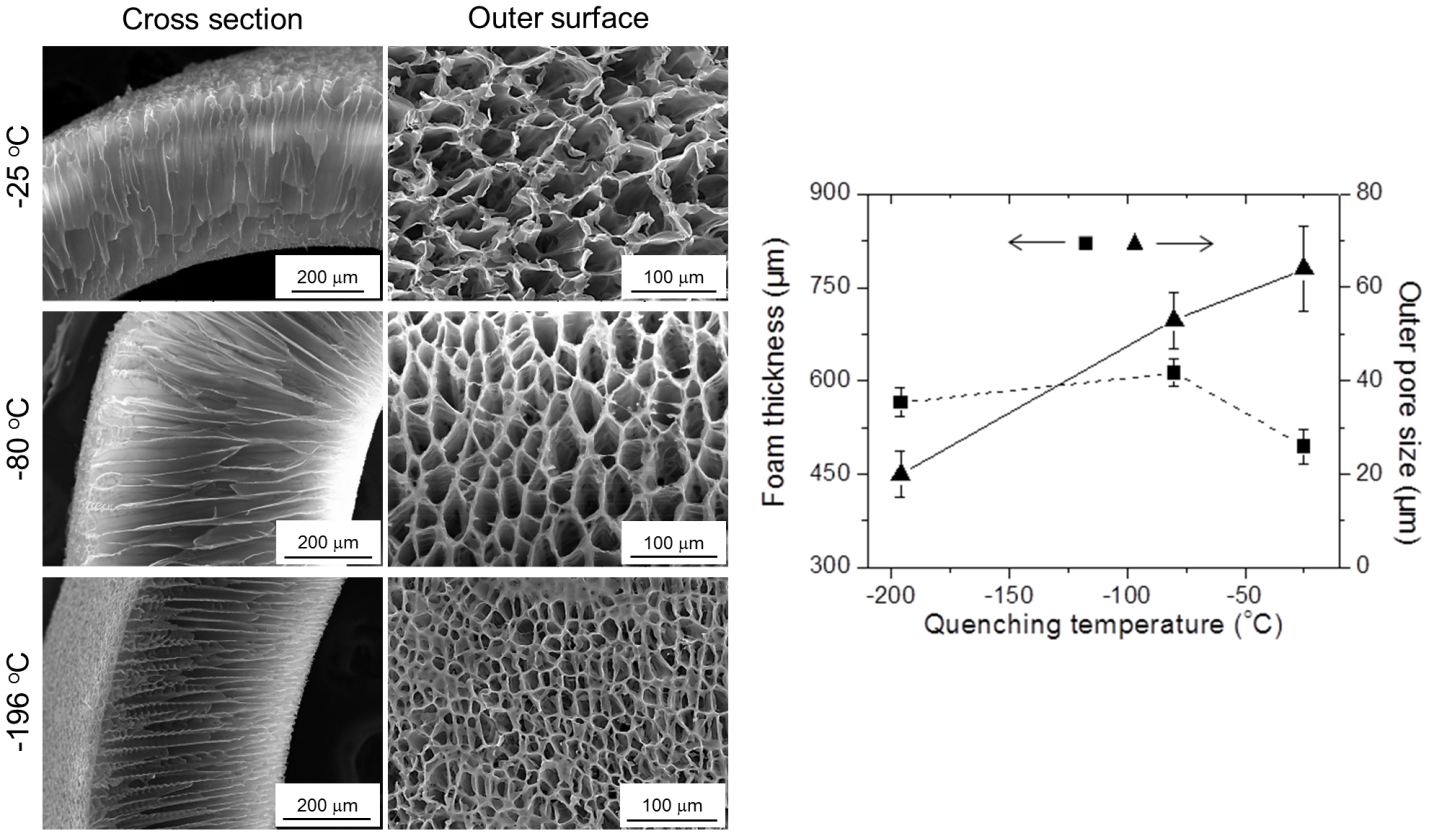
Influence of the quenching temperature. SEM images of the cross section and the outer surface of PLCL300 foams (5% w/v) prepared at various quenching temperatures (−25, −80 and −196°C) for 30 s revealed that the increase in the quenching depth promoted the organization of pores with a significant decrease in the average pore size, but with a slight alteration in the overall foam thickness. Measurements were done by *Image J* (*n* = 25). The differences in the foam thickness or the pore size between any two groups were found to be statistically significant. The correlation coefficients between the quenching temperature and the outer pore size and the foam thickness were calculated to be −0.99 and +0.42, respectively.

The ability to control the solvent crystallization is crucial because the crystallite geometry serve as a template for macro and micro structure of the pores. We investigated and compared the influence of three different quenching temperatures (all lower than the melting point of 1,4-Dioxane) on the pore architecture of PLCL300 foams with a constant polymer concentration (5% w/v). SEM images of the outer surface revealed that the average pore sizes of the foams quenched at −25, −80 and −196°C were 64±9, 53±6 and 20±5 µm, respectively (Figure 6). There was a clear indication of gradual reduction in the outer pore size with decreasing quenching temperature or, in other words, with increasing quenching depth. The overall foam thickness increased from 494±28 to 612±22 µm when the quenching temperature decreased from −25 to −80°C. However, further decrease in the quenching temperature causes only a slight change in the thickness (565±23 µm, at −196°C). The PLCL300 solution subjected to a higher temperature (−25°C) exhibited slow crystallization and an active phase coalescence by decreasing interfacial energy, and resulted in bigger pores. While, the solution subjected to lower temperature (−196°C) underwent fast crystallization and arrest of phase nucleation, and hence resulted in relatively smaller pores. These observations correspond with the data reported in the scientific literature [33], [35], [32], [39]. The foams prepared at −80°C showed a well-developed honey-comb shaped pore structure; therefore the cooling temperature of −80°C was the fixed process parameter for the following tests.

#### Quenching time

**Figure 7:**
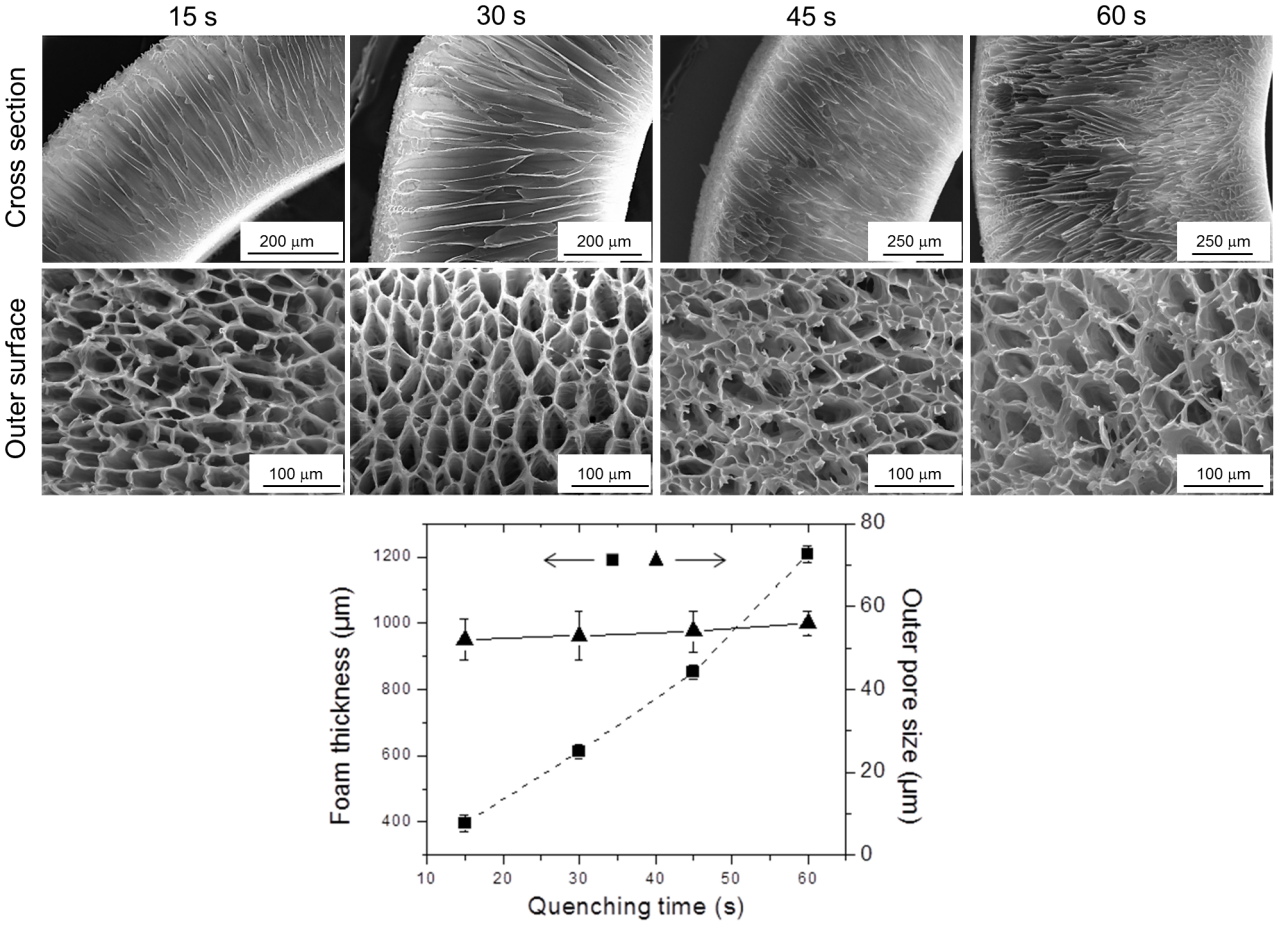
Influence of the quenching time. SEM images of the cross section and the outer surface of PLCL300 (5% w/v) foams prepared at −80°C for different quenching times (15, 30, 45 and 60 s) demonstrated the facile fabrication of foams of variable thickness without any significant change in the average pore size but with a slight thickening of the pore wall. Measurements were performed by *Image J* (*n* = 25). The differences in the outer pore sizes were statistically not significant, but the differences in the thickness between any two groups were statistically significant. The correlation coefficients between the quenching time and outer pore size and foam thickness were calculated to be +0.98 and +0.99, respectively.

The influence of four different quenching times (15, 30, 45 and 60 s) on the pore morphology of PLCL300 foams (5% w/v, in 1,4-Dioxane, quenched at −80°C) was assessed. The SEM micrographs of cross section and outer surface morphologies of the foams quenched for various time intervals are presented in Figure 7. The average thickness of the phase separated foams fabricated with the quenching times of 15, 30, 45 and 60 s were found to be 395±25, 612±22, 852±20 and 1208±26 µm, respectively. Such gradual increase in the polymer foam thickness could be attributed to the gradual increase in the amount of the phase separated polymer over the mold with the increasing quenching time. However, since there was no change in either the polymer concentration or the applied quench depth, there was a negligible effect on the mean outer pore sizes of the foams prepared at the selected quenching times. The increasing trend in the outer pore size at higher quenching times may be attributed to the decreasing gradient of quench strength from the source point (*template*) to the end point (the outer surface of the foam) [24], . Besides, as a matter of fact, the physical examination of the foams suggested the improved mechanical toughness due to the increased foam thickness. Overall, it was evident that merely by changing the quenching times, the current Dip TIPS method enabled the fabrication of foams with a variable thickness while maintaining the outer pore size, thus avoiding any adjustments in the setup (e.g. the size of the mold).

#### Coarsening time

**Figure 8:**
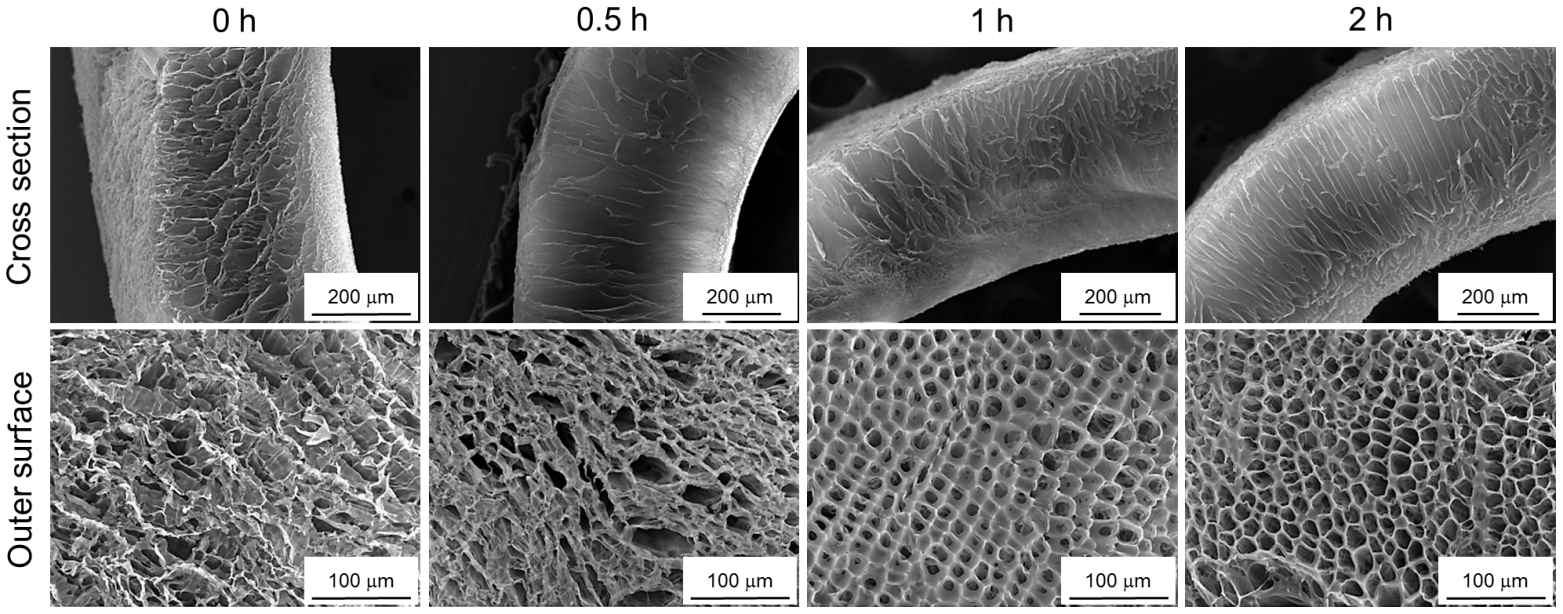
Influence of the coarsening time. SEM images of the cross section and the outer surface of the PLCL300 (3% w/v) foams prepared by quenching at −25°C for 30 s and coarsening for various times (0, 0.5, 1 and 2 h) suggested that an increase in the coarsening time resulted in better organization of the solvent rich phase what after solvent removal led to well-ordered pores.

The morphology evolution of the PLCL-dioxane binary phase systems in relation to increasing coarsening time was also investigated. The SEM micrographs showing the pore morphology of the PLCL300 scaffold (3% w/v, quenched at −25°C for 30 s) coarsened for 0, 30, 60 and 120 min) are presented in Figure 8. The observations clearly demonstrated that the foams with longer coarsening times yielded the well-ordered pore architecture in comparison with the foams coarsened for a shorter time. However, when foams were prepared from higher polymer concentrations (i.e., 5, 7 or 10% w/v) or at lower quenching temperatures (−80 or −196°C), no significant changes in the pore morphology were observed in relation to the coarsening time (data not presented). Our observations agree with the spinodal phase separation theories of polymer solutions, wherein it was suggested that under lower quench depth and lower polymer concentration, even though the initial spatial configurations of the phases were nearly random shortly after quenching, an anisotropic system develops strong spatial correlations along the elastically soft crystallographic directions of the phases during the subsequent coarsening. Thus, as the coarsening continues the system undergoes infinitesimal alignment to minimize the total free energy and yield evolved micro-structure of the phases [41]–[43]. On the other hand, the system undergoes instant crystallization and arrest of the phases under a higher quench depth. While in the case of higher polymer concentrations, the lack of a sufficient solvent phase leaves no scope for further fine tuning of the phases.

### Influence of the mold diameter on pore architecture

**Figure 9:**
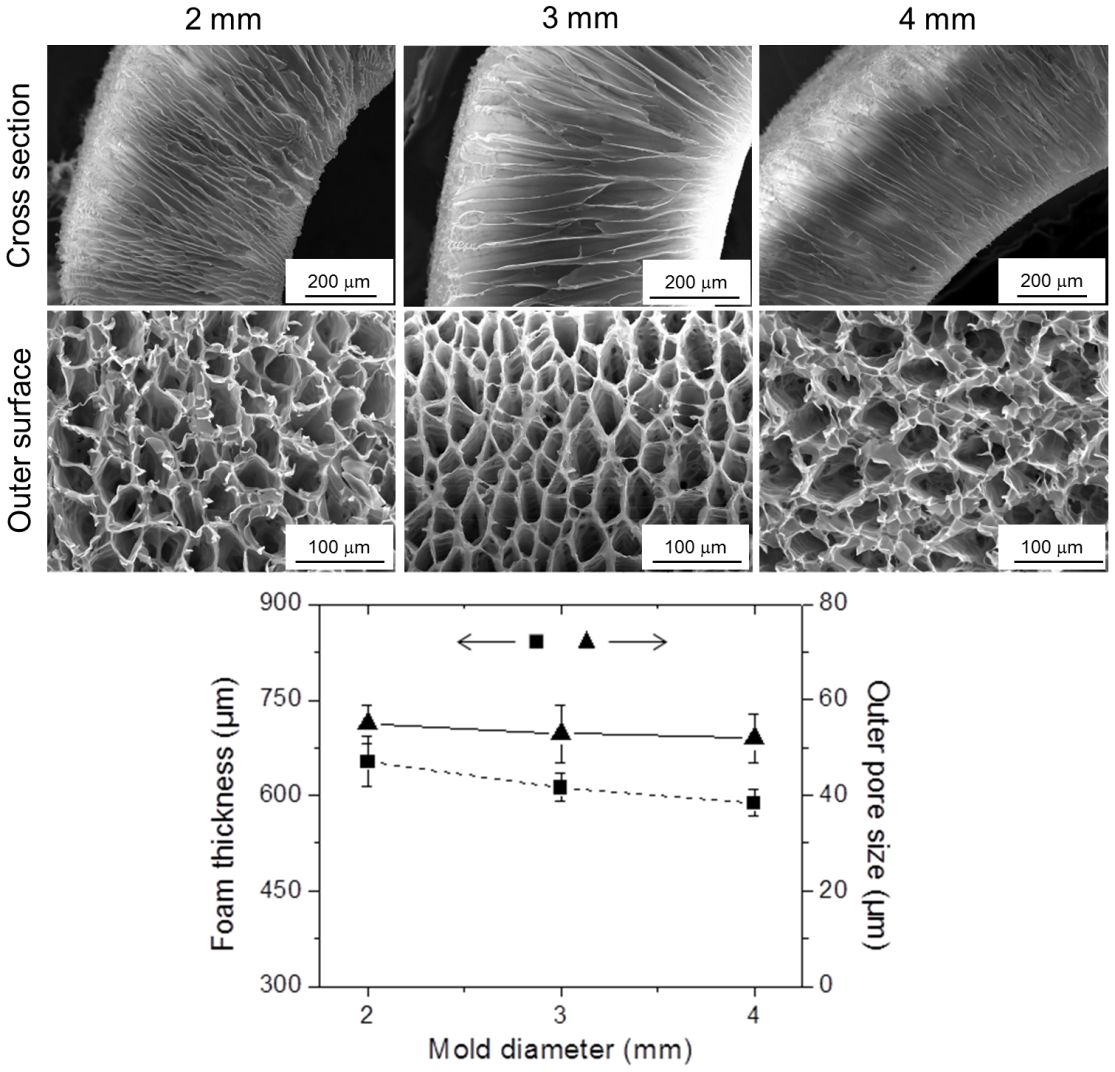
Influence of the mold diameter. SEM images of the cross section and the outer surface of the PLCL300 (5% w/v) foams prepared with molds of 2, 3 and 4 mm diameter at −80°C for 30 s demonstrated the ability to fabricate foams with variable inner lumen diameters without any significant change in the average pore size but with a slight thickening of the pore wall and decrease in the overall foam thickness. Measurements were performed by *Image J* (*n* = 25). The differences between any two groups were statistically not significant. The correlation coefficients between the mold diameter and outer pore size and foam thickness were calculated to be −0.92 and −0.98, respectively.

There were two metallic components in the Dip TIPS setup (Figure 1), i.e. the *conductor* with defined dimensions and the *template* of variable shapes and sizes. Hypothetically, any significant change in the *conductor* block would cause alterations in the final quench strength and thereby influence the resultant foam structure. However, since the *conductor* dimensions were predefined and kept unchanged for all the investigations, we have not explored this parameter in the current study. Instead, we investigated the effect of diameter of the *template* (2, 3, and 4 mm) on the final pore architecture. The morphological observations are presented in Figure 9. The formation of anisotropic oriented interconnected channeled pores was successful in all cases. As evident from the images, the increase in the mold diameter from 2, 3 to 4 mm lead to a slight decrease in the overall foam thickness from 653±39, 612±22 to 588±21 µm respectively. On the other hand, there was a small increase in the average pore size and the pore wall thickness. This can be attributed to the fact that, in the case of a lower diameter mold, the quench strength was plausibly higher and hence led to the increased phase separation of the polymer over the *template* accounting for the increased foam thickness. Also, perhaps the quench rate was quicker in the case of a lower diameter *template* and thus resulted in relatively smaller pores. However, the increase in the pore wall thickness with an increasing *template* diameter may be attributed to the possible spinodal phase separation of the solvent from the polymer phase associated with lower quench strength and slower quench rate.

### Thermal characteristics of PLCL300 foams

The aim of the DSC analysis was to characterize thermal properties of the PCLC300 foams (5% w/v) and to follow possible changes in the polymer crystallinity during the foam processing potentially induced by change in the quenching temperature. It was hypothesized that higher quench strength would yield lower crystallinity due to a rapid arrest of polymer and solvent phases, and vice versa. The results obtained for dried polymer foams are presented in Table 2; In general, there were no significant changes in the studied thermal characteristics for quenching temperatures −25, −80 and −196°C, and all tested samples showed a crystallinity of ∼30%. However, the melting temperature (*T*_m_) of the PLLA part from the PLCL300 copolymer and overall crystallinity of the processed foams exhibited a decreasing trend with respect to the increasing quench depth. Such trend was not observed in the second run due to the erase of thermal history. A similar behavior was observed by *Goh* and *Ooi* during the phase separation of a pure poly(l-lactide) (PLLA) by quenching the polymer solution in dioxane from room temperature to −25 or −80°C. No significant difference in the PLLA crystallinity before and after processing was observed (∼18%), although a slight crystallinity decrease with respect to increasing quench depth was detected [32]. The PLCL300-dioxane system undergoes the solid-liquid phase separation when quenching temperatures are much lower than the melting point of dioxane and *T_g_* of the polymer. Under such conditions, there was no opportunity for the polymer to undergo further crystallization during the foam processing [33], [32]. On the other hand, the decreasing trend in the crystallinity with respect to the decreasing quenching temperature could be attributed to the fact that the nucleation and growth of the PLCL crystallites at lower quench depth (e.g. −25°C) was reduced to a lower extent than in the case of systems prepared at higher quench depth (−80°C, −196°C) [32], [44].

**Table 2:** DSC analysis of PLCL300 before processing (control) and PLCL300 foams (5% wt, 30 s) prepared under various quenching temperatures (n = 3).

Quenching temperature	1^st^ run#			2^nd^ run					
	*T*_m_Δ*H*_f_ *X*_c_			*T*_g_ *T_c_*Δ*H*_c_ *T*_m_Δ*H*_f_ *X*_c_					
	(°C)	(J/g)	(%)	(°C)	(°C)	(J/g)	(°C)	(J/g)	(*_%_*)
Control*	164.1	31.1	34	30.2	84.9	−19.1	163.4	31.4	34
−25°C	162.3	31.4	34	33.5	89.0	−18.0	163.3	30.8	33
−80°C	163.0	29.1	32	34.9	90.6	−18.3	163.4	30.9	33
−196°C	162.5	28.6	31	31.3	92.8	−19.1	163.4	31.5	34

# the cold crystallization peak (*T_c_* and corresponding Δ*H_c_*) was not observed in the first run; also, the *T_g_* was not calculated in the first run.

* After polymer isolation.

### Porosity and surface area properties of PLCL300 foams

The aim of porosity studies was to characterize the porosity and surface area properties of the PCLC300 foams used for *in vivo* study and to follow possible changes in the porosity due to the change in the quenching temperature. The obtained data for the PLCL300 (5% w/v) foams prepared by quenching for 30 seconds at −25, −80 and −196°C are presented in Table 3. All the PLC300 foams were found to have a high porosity of about 90% which was comparable with the porosity of pure PLLA and PCL foams prepared by the standard TIPS but analyzed mostly by gravimetric methods [26], [32], [33], [35]. The mean pore diameter observed was ∼25 µm and the specific surface area varied between 4.4–4.9 m^2^/g. The foam porosity and the mean pore diameter had decreasing trend whereas the specific surface area increased with increasing quench depth. However, the changes observed were minimal and were considered as insignificant. In contrast, Guarino *et al*. [33] observed almost a doubled increase in the mean pore size for the PCL foams (5% wt, dioxane) from 25 to 50 µm when quenching temperature increased from −18 to 4°C. In the case of PLA foams prepared by TIPS, the pore size was characterized mainly by the microscopic methods (as also performed in the present study) and not giving the information about the mean pore size [24], [26], [32], [40]. As expected, the determined pore volume was relatively high (7–8.5 ml/g, Table 3) and reflected the pore diameter values together with high porosities. The effect of the quenching temperature was prominent over the pore volume; the pore volume distinctively decreases with decreasing quenching temperature in accordance with decreasing trends in the porosity and the mean pore size.

**Table 3:** Mercury intrusion porosimetry and BET surface area analysis of PLCL300 foams (5% wt, 30 s) prepared at various quenching temperatures (n = 3).

Quenching temperature	Mean pore diameter (µm)*	Pore volume (ml/g)	Porosity (%)	BET surface area (m^2^/g)
−25°C	26	8.5	90	4.4
−80°C	25	7.1	88	4.8
−196°C	23	7.0	87	4.9

* Pore range considered: 0.2 and 116 µm.

**Figure 10:**
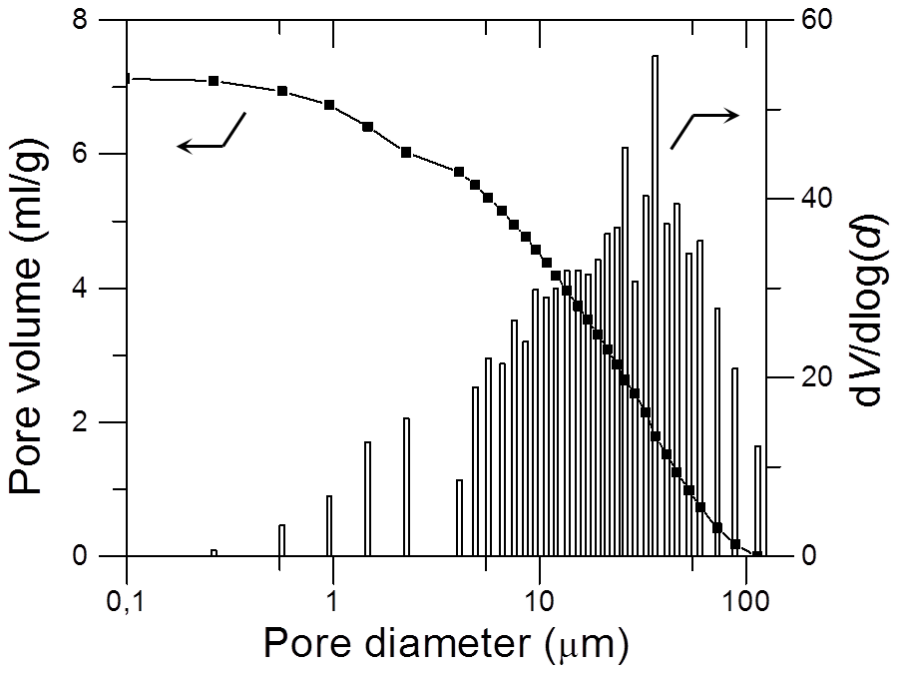
Mercury porosity analysis. The cumulative pore volume (line with markers) and derivative pore size distribution (vertical columns) of PLCL300 foam prepared at −80°C for 30 s (pore range: 0.2 to 116 µm).

Figure 10 shows a typical cumulative pore volume curve and a derivative pore size distribution observed for the analyzed PLCL300 foams. Generally, the pores are characterized by a broad distribution that can be attributed to strongly anisotropic character of the channeled pores observed by SEM (Figure 2); the determined pore size distribution (expressed in a logarithm scale as the d*V*/dlog(*d*) ratio) ranged from 0.2 to 116 µm and the pores with the size from 10 to 70 µm were found to be the most frequent. The mean pore size calculated by the porosimetry is not comparable with the outer and inner pore size obtained by the SEM analysis; the porosimetry analysis considered for calculations all the pores ranging from 0.2 to 116 µm throughout the foam, whereas SEM calculations were performed by considering the pores visible on the outer surface only. As a matter of fact, the gas/fluid intrusion based methods solely allow the evaluation of the open-porous and interconnected network and do not yield any results in case of the closed-porous foams [33], [45]. Thus, the determined 90% porosity for PLCL300 foams straightway depicts a highly interconnected open porous nature of the foams fabricated by Dip TIPS. This feature of the foam morphology is an essential parameter for the successful colonization of foams by the cells and formation of the tissue in the *in vivo* conditions [3]. However, the data obtained from porosimetry analysis did not enable to deduce a correlation between Dip TIPS process parameters and pore parameters. The data obtained was not significant enough in this regard. Thus, we followed the SEM-based pore size analysis to deduce the possible correlation (in earlier sections).

### *In vivo* behavior of PLCL300 foams

**Figure 11:**
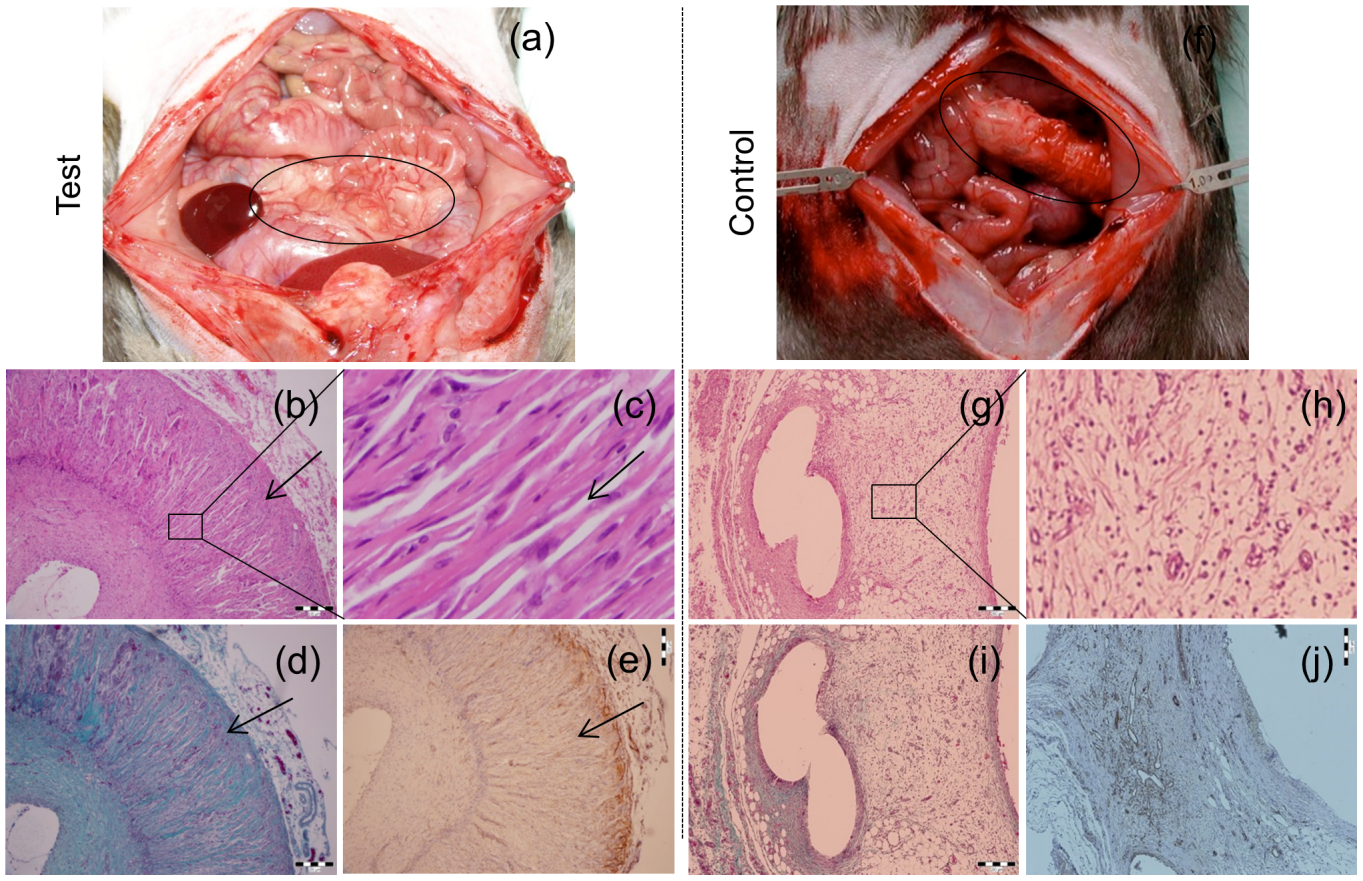
*In vivo* behavior of the test (a–e) and control (f–j) scaffolds. (a, f) 4-week old anastomosed scaffolds as seen in the omentum of the model animal; (b, g) low magnification and (c, h) high magnification H&E stained sections showing the host cell infiltration and cell distribution (deep blue-purple nuclei, pink cytoplasmic and extracellular proteins); (d, i) TRI stained sections showing the synthesis of extracellular collagen (blue or green) by the invading cells; and (e, j) anti-CD31 stained sections showing the infiltration of microvascular endothelial cells (brown). The black arrow in the panels (b–e) represents the direction of host cell/tissue infiltration. Scale: 200 µm.

Since polylactide products are biocompatible and biodegradable polymers that have long been approved by the United States Food and Drug Administration for use in medical applications [46], we did not consider re-establishing its biocompatibility in general. However, based on our previous experiences, the implanted scaffolds can induce a temporary fluid accumulation and mechanical irritation of surrounding tissue [11]. To this end, the commonly performed *in vitro* studies do not allow a proper cell ingrowth into the scaffold, especially under static conditions [15]. Therefore, in the current study, we have studied the influence of the scaffold architecture on the cell infiltration, extracellular matrix synthesis and vascularization *in vivo* using a rat animal model. Accordingly, the PLCL300 foams (5% w/v in dioxane, quenched at −80°C for 30 s) and the control macroporous scaffolds (made from monofilament polydioxanone fibers, by knitting) were implanted into the greater omentum of healthy brown Norway rats. The scaffolds were engrafted by a gentle fibrous membrane (including vessels) in one week and did not cause any side effect such as inflammation or an excessive fibrotic reaction (data not presented). After 4 weeks, all the rats with the PLCL300 survived the procedure and were no different from the controls. The implants were excised and analyzed for the basic morphology (H&E staining), production of fibrous tissue (TRI staining) and the presence of endothelial cells (CD31 staining) representing mature as well as newly formed blood capillaries. The results are presented in Figure 11. Based on the macroscopic observations, both the tested PLCL300 and the control macroporous scaffolds maintained their geometrical shape and structural integrity during the implantation, engraftment and excision (Figure 11a, 11f).

The H&E staining provides information about the general morphology of samples under examination. The cross section images of the H&E stained samples of the PLCL300 foams depicted the guided infiltration of the cells coming from the surface to the center and the homogenous distribution of the cells throughout the foam (Figure 11b). A high magnification image clearly revealed a guided infiltration of the host cells through the oriented channeled pores of the microporous PLCL300 scaffolds (Figure 11c). On the other hand, the fibrous tissue was randomly spread across the control macro-porous scaffold (Figure 11g, 11h). The TRI staining usually produces blue or green collagen, light red or pink cytoplasm and dark brown or black cell nuclei. The TRI stained cross section analysis showed the active synthesis of extracellular matrix components, particularly collagen, by the invading host cells (preferentially the cells of the connective tissue) in the PLCL300 foam (Figure 11d); in contrast, the macroporous scaffold analysis revealed a poor matrix synthesis by cells (Figure 11i). Apart from the cell infiltration and extracellular matrix synthesis, another important factor required for the successful tissue regeneration is the scaffold's ability to support angiogenesis. The CD31 staining is a widely used immuno-histochemistry method to demonstrate the presence of vascular endothelial cells that have an abundant CD31 surface marker. In the current study, the CD31 stained cross section images showed that not only the fibroblast-like cells but also the vascular endothelial cells and microvascular network (Figure 11e, brown colored) were present on the surface of the implant; however, they did not migrate towards the internal surface of the scaffold. On the other hand, the control scaffold showed the scattered presence of endothelial cells (Figure 11j).

Overall, the preliminary *in vivo* investigations showed that the anisotropic radially oriented channeled pores of adjusted pore-size parameters successfully offered the guidance for the host cell infiltration and provided convenient conditions (for exchange of nutrients, gases, secretions, etc.) for supporting higher cell density and uniform cell distribution. Importantly, there was no sign of any fibrous capsule formation around the implant, thus suggesting the compatibility of the implant material with the surrounding tissue. Our results correlate with the findings in several earlier investigations using, for example, the PLA scaffold with aligned channeled pores prepared by super critical fluid processing [47], or PLA scaffolds with the anisotropic channeled pores [24], as well as the chitosan scaffold with uniaxial tubular pores prepared by TIPS [48].

### Features of Dip TIPS

The polymer foams with anisotropic interconnected channeled pores are increasingly gaining importance in the guided tissue engineering and other related applications. However, till now the preparation of anisotropic channeled porous foams by TIPS was done by using complex setups [24]–[26], [33], [40], [48], [49]. For example, to prepare PLA tubular foams with lumen diameters up to 3 mm and the foam thickness from 1 to several millimeters, the molds were assembled by inserting a thermally conductive cylindrical *template* into a thermally non-/less- conductive capillary tube [24], [49]. To obtain a uniform foam thickness, the *template* was held tightly within the capillary tube by tapes or sleeves at both ends [49]. The fabrication of PLA tubular foams for nerve regeneration with a variable inner lumen diameter (>3 mm) or wall thickness (>0.5 mm) involved complex adjustments including additional metal molds in the setup [49]. For the construction of flat 3D foams, the molding setup included a tailored thermally non-/less- conductive container with single (at bottom) or double (at top and bottom) plate(s) of high thermal conductivity [33]. Hence, in general, the successful fabrication of anisotropic foams by the conventional TIPS setups described above requires a number of accessories, different setups for preparation of differently shaped foams and skillful hands for careful and precise maneuver.

**Figure 12:**
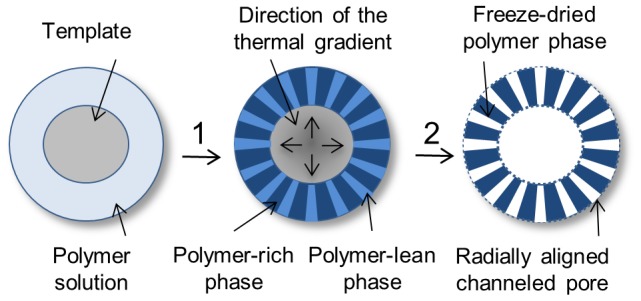
Dip TIPS working principle. A schematic illustration of the mechanism of the formation of anisotropic oriented interconnected channeled pores (1: quenching, 2: freeze-drying).

In the current study, we demonstrate a process termed as Dip TIPS that fundamentally relies on the principle of unidirectional TIPS (Figure 12). When the *template* that was dipped in the polymer solution was subjected to quenching using a cooling mixture, the polymer solution in the immediate vicinity of the *template* has underwent unidirectional phase separation along the direction of thermal quenching [21], [35]. In Dip TIPS, similar to the conventional TIPS, the phase separation proceeds through either the solid-liquid or the liquid-liquid phase separation phenomenon depending on the applied quenching depth and composition of the system [33], [35], [32]. However, in contrast to the previous methods, the Dip TIPS methodology allowed the fabrication of tubular, open-end capsular and flat 3D foams with a variable pore size, foam thickness and inner lumen diameters, without complex adjustments. A summary of the results obtained is presented in Table 4. A comparison of Dip TIPS with the state of the art methods for the fabrication of anisotropic porous foams of various shapes and sizes with controlled pore architecture is presented in Table 5.

**Table 4:** Summary of the effects of various process parameters on the foam properties (n = 6).

Parameter studied	Observations
Polymer type (PLA, PCL, PLCL)	Pore size: PLA<PLCL<PCL; Foam thickness: PLA>PLCL>PCL; PLA foams were rigid and brittle with prominent micro-cracks. PCL foams were soft and elastic with collapsed pore architecture upon manipulations. PLCL foams were tough and without micro-cracks.
Polymer molecular weight (PLCL150, PLCL300)	Pore size: PLCL150>PLCL300; Foam thickness: PLCL150<PLCL300; The PLCL300 foams had consistently well-organized pore structure with significantly thicker pore walls and enhanced toughness in contrast to the PLCL150 foams
Polymer concentration (3, 5, 7 and 10% w/v)	Pore size: 3>5>7>10; Foam thickness: 3<5<7<10; In contrast to the foams prepared from concentrated PLCL solution (5% or higher), the foams from 3% exhibited irregular and undefined pore architecture. A gradual thickening of pore walls in a direct correlation with the polymer concentration was also observed.
Quenching temperature (−25, −80, −196°C)	Pore size: −25>−80>−196; Foam thickness: −25<−80<−196; The physical examination revealed that the mechanical strength of the capsules increased with an increasing quench depth.
Quenching time (15, 30, 45, 60 s)	Pore size: no significant changes; Foam thickness: 15<30<45<60; An increase in the quenching time led to the thickening of the foam that in turn led to enhanced mechanical strength (as per the physical examination).
Coarsening duration (0, 30, 60, 120 min)	The effects observed only for 3% (w/v) PLCL300 solution quenched at −25°C.
Mold diameter (2, 3, 4 mm)	Pore size: no significant changes; Foam thickness: no significant changes; A gradual thickening of pore walls in a direct correlation with the mold diameter was noted.

**Table 5:** Summary of the practical advantages of Dip TIPS in comparison with the state of the art method for the fabrication of anisotropic porous foams of various shapes for various applications.

Foam type	Potential applications	State of the art methodology	Dip TIPS methodology
Porous open-end capsular foams	Cell transplantation	No method reported till now	The setup involves a set of easy-to-assemble *template*, *conductor* and *reservoir*, and enables the fabrication of open-capsules with variable inner lumen diameter, wall thickness and with controlled pore architecture.
Porous tubular foams	Vascular, nerve or other tubular tissue engineering	Typical setup [24], [49]. Molds contain one inner metallic bar and one outer non-metallic cylinder with helical locks at both ends, or with metallic bottom; requires another setup with appropriate dimensions for controlling both lumen diameter and foam thickness. the outer surface is often prone to the skin-effect with low/no porosity.	Post-fabrication cutting of the closed end of the open-capsule leads to a tubular foam; fabrication of tubular foams with a variable inner lumen diameter is achieved by a simple exchange of the *template* of a desired size; fabrication of tubular foams with a variable thickness is possible by a mere change in the quenching time.
Porous flat foams	Guided tissue engineering	Typical setup [14], [33]. Molds contain a dish with metallic bottom but with non-metallic walls; obtaining homogenous foam thickness, especially in the case of low thickness foams, is difficult due to concave meniscus of the polymer solution in the mold; the outer or top surface is often prone to the skin-effect with low/no porosity.	The same mold as for open-capsular/tubular foams, except the use of a flat *template* instead of a cylindrical one; the thickness of flat foams is nearly homogenous; preparation of flat foams with a variable thickness is possible by a mere change in the quenching time; there is skin-effect, but, it is not a process related error, rather it is a manual error (such as the exposure of the just phase separated frozen sample to environment, due to a delay during the transfer from the conductor to a freezer or freeze drying).

The conventional setups follow the thermal conductivity directly from the primary source, while Dip TIPS follows the thermal conductivity from the extended surface of the primary source (known as the “fin”). Hence, the heat transfer principles governing the conventional and Dip TIPS are different. A detailed description of these mechanisms has been described elsewhere [50], [51]. In brief, the fins on the base surface are made either by extrusion, welding or simple mechanical fixing to enhance or extend the heat transfer from a given surface. The thermal conductivity properties and dimensions of the fin as well as the resistance in the joint between the primary source and the fin determine the thermal profile [50], [51].

In the context of Dip TIPS, the shape, size and material composition of the fin used affects the formation of the foams and the properties there-of. In the current study, cylindrical and rectangular Dural fins with a thickness of 3 mm and a length of 40 mm were used; nearly uniform thermal profile (indicated by a nearly-uniform foam thickness for a foam length of 15 mm) was observed. Further, the solution properties, in particular the solvent properties profoundly affect the initial nucleation process and homogeneity of the phase separation along the length of the fin. In our experience in this regard, we observed that the organic solvents such as dioxane was readily able to undergo nucleation, but it was difficult to achieve in the water system; This could be attributed to differences in the enthalpy of crystallization of various solvents or solvent systems. Thus, although the presented Dip TIPS setup can be in principle applied for the controlled preparation of any polymer foams, we anticipate that the fin, polymer and solvent properties could potentially influence the foam formation and its properties.

## Conclusions

We demonstrated a facile methodology termed as “Dip TIPS” for the fabrication of polymer foams with anisotropic interconnected channeled pores with an ascending gradient pore size perpendicular to the mold. The method works on the principle of unidirectional TIPS of a polymer solution that involves the separation of the polymer and solvent phases along the direction of the thermal gradient. In comparison to other complex methods, the process readily enabled the fabrication of PLA based foams in shapes such as tubules, open-end capsule and flat 3D sheets. The foams with thickness between 395 and 1208 µm were obtained merely by changing the quenching times (15, 30, 45 and 60 s). The foams with inner lumen diameters of 2, 3 and 4 mm were obtained easily by changing the mold diameter. On the other hand, the pore size (between 20 to 65 µm) was controlled by changing either the quenching temperature (−25, −80 and −196°C) or the polymer concentration (3, 5, 7 or 10% w/v). The current study confirmed the previously published data that suggested an inverse relation between the pore size, and the concentration and molecular weight of the polymer, and a direct relation between the pore size and the applied quenching temperature. The preliminary *in vivo* investigations in brown Norway rats showed that the selected PLCL300 scaffolds were biocompatible as there was no inflammation or an excessive fibrotic reaction observed and that the character of the pore structure supported the guided cell infiltration and homogenous cell distribution within the scaffolds in comparison to the control macroporous scaffold. Further studies focused on the enhancement of the surface morphology of the scaffold, the encapsulation of bioactive factors and the subsequent evaluation of its *in vivo* potential are in progress.
